# Supplementary material for: Single-cell characterization of anterior segment development in the mouse reveals the cell types, pathways, and signals driving formation of the trabecular meshwork and Schlemm’s canal
Source: eLife. 2026 Jul 21;15:RP109230. doi: 10.7554/eLife.109230 (PMC13387745; doi:10.7554/eLife.109230)
Supplement: Peer review history

# Editor report: eLife Assessment

**DOI:** 10.7554/eLife.109230.3.sa0

**Authors:** Moussa Zouache

**Affiliations:** University of Utah United States

**Keywords:** Compelling, Important

This **important** work advances our understanding of the development of the visual system. The data presented is **compelling** and provides a detailed single-cell atlas of post-natal anterior chamber development in mice, highlighting the trabecular meshwork and Schlemm's canal.

# Referee report: Reviewer #2 (Public review)

**DOI:** 10.7554/eLife.109230.3.sa1

Summary:

This study presents a detailed single-cell transcriptomic analysis of the post-natal development of mouse anterior chamber tissues. The dataset is robust, consisting of ~130,000 cells collected across seven time points from early post-natal development to adult. Analysis focused on the development of cells that comprise Schlemm's Canal (SC) and trabecular meshwork (TM).

Comments on revisions:

My critiques have been adequately addressed.

# Author comment: Author response

**DOI:** 10.7554/eLife.109230.3.sa2

**Authors:** Revathi Balasubramanian, Nicholas Tolman, Taibo Li, Abdul Hannan, Violet Bupp-Chickering, Karina Polanco, Aakriti Bhandari, Sally Zhou, Marina Simón, John Peregrin, Christa Montgomery, Krishnakumar Kizhatil, Jiang Qian, Simon WM John

**Affiliations:** Columbia University Irving Medical Center New York United States; Columbia University Irving Medical Center New York United States; Johns Hopkins University School of Medicine Baltimore United States; Columbia University New York United States; New York University New York United States; Columbia University Irving Medical Center New York United States; University of Utah Salt Lake City United States; SUNY Downstate Health Sciences University New York United States; Columbia University Irving Medical Center New York United States; Columbia University Irving Medical Center New York United States; Columbia University Irving Medical Center New York United States; The Ohio State University Wexner Medical Center Columbus United States; Johns Hopkins Medicine Baltimore United States; Columbia University Irving Medical Center New York United States

The following is the authors’ response to the original reviews.

**Public Reviews:**

**Reviewer #1 (Public review):**

Summary:

This study presents a comprehensive single-cell atlas of mouse anterior segment development, focusing on the trabecular meshwork and Schlemm's canal. The authors profiled ~130,000 cells across seven postnatal stages, providing detailed and solid characterization of cell types, developmental trajectories, and molecular programs.

Strengths:

The manuscript is well-written, with a clear structure and thorough introduction of previous literature, providing a strong context for the study. The characterization of cell types is detailed and robust, supported by both established and novel marker genes as well as experimental validation. The developmental model proposed is intriguing and well supported by the evidence. The study will serve as a valuable reference for researchers investigating anterior segment developmental mechanisms. Additionally, the discussion effectively situates the findings within the broader field, emphasizing their significance and potential impact for developmental biologists studying the visual system.

Weaknesses:

The weaknesses of the study are minor and addressable. As the study focuses on the mouse anterior segment, a brief discussion of potential human relevance would strengthen the work by relating the findings to human anterior segment cell types, developmental mechanisms, and possible implications for human eye disease. Data availability is currently limited, which restricts immediate use by the community. Similarly, the analysis code is not yet accessible, limiting the ability to reproduce and validate the computational analyses presented in the study.

In the revised version we have added an additional paragraph to the discussion section highlighting the human relevance of our work. Additionally, data is public on single cell portal and GEO, accession numbers have been updated. Codes are available on Github (https://github.com/revathi-balasubramanian/Anterior-segment-development-single-cell-data-analysis).

**Reviewer #2 (Public review):**

Summary:

This study presents a detailed single-cell transcriptomic analysis of the postnatal development of mouse anterior chamber tissues. Analysis focused on the development of cells that comprise Schlemm's Canal (SC) and trabecular meshwork (TM).

Strengths:

This developmental atlas represents a valuable resource for the research community. The dataset is robust, consisting of ~130,000 cells collected across seven time points from early post-natal development to adulthood. Analyses reveal developmental dynamics of SC and TM populations and describe the developmental expression patterns of genes associated with glaucoma.

Weaknesses:

(1) Throughout the paper, the authors place significant weight on the spatial relationships of UMAP clusters, which can be misleading (See Chari and Patcher, Plos Comb Bio 2023). This is perhaps most evident in the assessment of vascular progenitors (VP) into BEC and SEC types (Figures 4 and 5). In the text, VPs are described as a common progenitor for these types, however, the trajectory analysis in Figure 5 denotes a path of PEC -> BEC -> VP -> SEC. These two findings are incongruous and should be reconciled. The limitations of inferring relationships based on UMAP spatial positions should be noted.

(2) Figure 2d does not include P60. It is also noted that technical variation resulted in fewer TM3 cells at P21; was this due to challenges in isolation? What is the expected proportion of TM3 cells at this stage?

(3) In Figures 3a and b it is difficult to discern the morphological changes described in the text. Could features of the image be quantified or annotated to highlight morphological features?

(4) Given the limited number of markers available to identify SC and TM populations during development, it would be useful to provide a table describing potential new markers identified in this study.

(5) The paper introduces developmental glaucoma (DG), namely Axenfeld-Rieger syndrome and Peters Anomaly, but the expression analysis (Figure S20) does not annotate which genes are associated with DG.

(1) We agree that inferring biological relationships from the spatial arrangement of UMAP clusters has limitations and we have qualified our interpretation accordingly in the text. We have also added clarifying language to the trajectory analysis in Figure 5. The intended developmental trajectory is PEC → VP → BEC and SEC; however, the cluster labels in Figure 5 were applied incorrectly. Specifically, VP, BECs cluster was mislabeled as BECs, which led to the confusion. This cluster contains VPs that transition into BECs as well as VPs that are precursors to SECs.

(2) We recently published the P60 dataset separately (Tolman, Li, Balasubramanian et al., eLife 2025); these data consist of integrated single-nucleus multiome profiles that were subjected to in-depth analysis. Additionally, we found that integrating the P60 dataset with the developmental datasets obscured sub-clustering of mature cell types. In future manuscripts, we will pursue a more detailed analysis of TM development and perform time point–specific clustering, similar to the approach we used for endothelial cells (Figure 4e).

Comparing proportions of cells at different ages and as the eyes grows needs to be done cautiously. Notwithstanding the limitations, the proportions of TM1, TM2, and TM3 clusters are expected to be similar between P14 and P21 as the proportions at P14 and P60 are similar when comparing to the separately analyzed P60 data. Importantly, our dissection strategy changed with age: from P2 to P14, we removed approximately one-third of the cornea, whereas at P21 and P60 we removed most of the cornea to help maximize representation of limbal cells as the eyes grew. This change in dissection likely contributed to the reduced number of TM3 cells observed at P21. TM3 cells are enriched anteriorly (at-least in adult) and so are located closer to the corneal cut during dissection of the P21 eyes (which despite being larger than younger ages are still small and more delicate to accurately dissect than at P60) and are therefore more likely to be lost. Additional details are provided in the Methods section and the caveats surrounding our dissection method have now been included.

(3) For Figure 3a and b, we have now pseudo-colored the spaces and provided a quantification of how both TM volume and intratrabecular spaces change with developing age (Figure 3c).

(4) We have now included a supplemental table of markers for developing and mature TM and SC cell types (Table S3).

(5) We have highlighted DG genes in rectangular boxes in Figure S20.
